# Relationship between Na/K-ATPase in thawed sperm and fertility of Angus bulls

**DOI:** 10.1590/1984-3143-AR2022-0066

**Published:** 2023-11-20

**Authors:** Juliana Carla Cavalcanti Marques, Allan Rodolf Ribeiro Cezar, Agnelo Douglas do Nascimento, Juliane Pereira da Silva, André Mariano Batista, Maria Madalena Pessoa Guerra, Diogo Ribeiro Câmara

**Affiliations:** 1 Laboratório de Reprodução Animal, Universidade Federal de Alagoas, Viçosa, AL, Brasil; 2 Laboratório de Biologia Celular, Universidade Federal de Alagoas, Maceió, AL, Brasil; 3 Laboratório de Andrologia, Universidade Federal Rural de Pernambuco, Recife, PE, Brasil

**Keywords:** ion channel, spermatozoa, transmembrane protein

## Abstract

Since bull fertility prediction remains challenging, the identification of potential fertility markers is important considering the economic benefits to the livestock industry. The main goal of this study was to determine the Na/K-ATPase activity and expression in thawed sperm of high (HF)- and low-fertility (LF) Angus bulls. Samples from three different batches/bulls with HF (n = 4) and LF (n = 4) were used. The Na/K-ATPase activity was determined after thawing, whereas sperm kinematics, membrane integrity, and expression of Na/K-ATPase on sperm surface were evaluated immediately post-thaw and after 120 minutes of incubation. Within the same incubation time, there was no difference on sperm membrane integrity, kinematics, and the expression of Na/K-ATPase on the sperm surface between HF and LF bulls. Kinematic parameters of LIN and VCL were not influenced by incubation time in samples from HF and LF, respectively. A tendency (P = 0.06) of higher Na/K-ATPase enzymatic activity for sperm of HF bulls compared to LF bulls was observed (0.49 ± 0.07 and 0.32 ± 0.06, respectively). In conclusion, Na/K-ATPase activity and expression in thawed sperm from Angus bulls are not related to the fertility index after fixed-time artificial insemination. However, sperm kinematics related to hyperactivation might indicate higher sperm cryotolerance for HF bulls.

## Introduction

To evaluate the reproductive potential of a sire, the traditional andrological evaluation is usually performed and able to identify infertile animals. However, this strategy can generally not distinguish between fertile and subfertile bulls, since thawed sperm within a minimal quality standard might result in approximately 20% of variation in the pregnancy rate (Kastelic and Thundathil, 2008; Pacheco et al., 2021). Moreover, in beef herds, the bull has a higher influence on reproductive failure than in dairy herds (Flowers, 2013), highlighting the importance of in-vitro fertility markers in beef bulls with further economic benefits to the livestock industry.

Seminal plasma protein and metabolic markers (Muhammad Aslam et al., 2014; Velho et al., 2018; Willforss et al., 2021; Klein et al., 2022), genetic analysis (Taylor et al., 2018; Pacheco et al., 2020), and sperm cell characteristics (Erickson et al., 2015; Kumaresan et al., 2017) are some of the potential targets to predict bull fertility. Among these potential markers, the relevance of Na/K-ATPase for sperm fertility have been studied in the last decades in different species; its influences sperm motility, capacitation, membrane potential, and pH (Blanco and Mercer, 1998; Woo et al., 2000; Kaplan, 2002; Newton et al., 2010; Jimenez et al., 2010, 2011; Lestari et al., 2017; McVey et al., 2018). The Na/K-ATPase is a transmembrane protein constituted of subunits differentially expressed among cells and tissues, e.g., subunit α1 is considered omnipresent in several tissues, whereas expression of α4 subunit is restricted to the male reproductive tract (Woo et al., 2000; Konrad et al., 2011; Syeda et al., 2020).

In Holstein bulls, the potential relationship between Na/K-ATPase concentration/activity and bull fertility has been reported (Rajamanickam et al., 2017b), as well as its physiological relevance in the regulation of some sperm features (Thundathil et al., 2018; Unnikrishnan et al., 2021). However, sperm parameter indicators of bull fertility are influenced by breed (Morrell et al., 2017, 2018), and studies investigating the influence of Na/K-ATPase on beef bull fertility are lacking. In this context, the goal of the present study was to determine the association between the fertility of beef bulls, previously classified as high (HF) and low fertility (LF) after fixed-time artificial insemination (FTAI), and the Na/K-ATPase content and activity. Sperm kinematics and membrane integrity were also assessed.

## Methods

Semen batches from eight proven sperm donors Angus bulls were kindly provided by a commercial AI center (Alta Genetics^®^; Uberaba, Brazil). After a field fertility test, with results of at least 500 FTAI per bull, they were classified as HF and LF after FTAI, which differed in approximately 8.0% (P = 0.029), with the standard deviation of the general fertility average ranging from 0.33 to 0.82 and - 2.52 to -1.71% for HF and LF bulls, respectively (Concept Plus Program, Alta Genetics^®^, Brazil). Three different batches from each bull used in the field fertility test (HF, n = 4, LF, n = 4) were evaluated in the present study, totalizing 24 semen batches. All samples were maintained in liquid nitrogen (-196°C) and thawed in a waterbath (37°C, 30 s) before analyses.

### Sperm kinematics and membrane integrity

Two straws from each batch/bull were thawed, and the contents were transferred to a microtube and homogenized. Sperm kinematics and membrane integrity were assessed immediately after thawing (0h) and following 120 min of incubation in a waterbath (37°C). The kinematic parameters were evaluated using the CASA system, and at least five nonconsecutive, randomly selected microscopic fields per sample were scanned, recording at least 500 sperm. Events not related to sperm were removed, and image sequences were saved and later analyzed. The following end points were analyzed: total motility (TM, %), progressive motility (PM, %), linearity (LIN, %), straightness (STR, %), wobble (WOB, %), curvilinear velocity (VCL, µm/s), progressive velocity (VSL, µm/s), path velocity (VAP, µm/s), amplitude of lateral head displacement (ALH, µm), and beat frequency of the tail (BCF, Hz). Motility end points were measured with the following settings: temperature, 37°C; frame rate, 25 s; minimal contrast, 75; frame number, 25 per field; sperm velocity that can be analyzed, 0 to 180 µm/s; and threshold STR, 75%.

Sperm membrane integrity was assessed using a commercial kit (LIVE/DEAD™ Sperm viability kit, Molecular Probes, Eugene, OR, USA; code L7011), according to the manufacturer’s guidelines. Aliquots (25 μL) of thawed semen samples from each batch/bull were diluted in modified TALP (NaCl 100 mM, KCl 3.1 mM, NaH_2_PO_4_ 0.3 mM, MgCl_2_6H_2_O 0.4 mM, Hepes 40 mM, Lactate 21.6 mM, PVA 0.1 mM; 37°C; 1:10, v:v) before the analysis. In total, 200 cells of each sample were evaluated under a fluorescence microscope (400× magnification; 450–490 nm excitation/emission filter; FWL- 3500 T FL, Feldmann Wild Leitz, Manaus, AM, Brazil); sperm with intact membrane were labeled in green and non-intact sperm membrane labeled in red.

### Na/K-ATPase activity

The method is a modification of that described by Rajamanickam et al. (2017b). Two straws from each batch/bull were thawed, the total contents were transferred to a microtube, and 500 µL of modified TALP was added, followed by centrifugation (500 *g*, 10 min, 8°C). After the supernatant was discarded, the sperm pellet was resuspended with 1 mL of extraction buffer (imidazole 30 mM, sucrose 250 Mm, NaEDTA 1 Mm) and homogenized. The homogenized content was split into two microtubes: the first with the sperm suspension on extraction buffer only and the second was ouabain-treated (sperm suspension on extraction buffer + ouabain 4 × 10^-4^ M) to inhibit Na/K-ATPase activity. The samples were kept under refrigeration (5 °C) for 60 min and vortexed (10 s) every 15 min. Subsequently, the samples were centrifuged (15,000 *g*, 15 min, 22°C), and the supernatant was recovered and diluted in Milli-Q water (1:20, v:v), before Na/K-ATPase activity measurement.

The Na/K-ATPase activity was determined in triplicate, using a commercial kit (Sigma-Aldrich, St. Louis, MO, USA; code MAK307) according to the manufacturer’s guidelines. The quantification of Na/K-ATPase activity was determined by calculating the difference in released phosphate (rP) between samples without ouabain and ouabain-treated. The Na/K-ATPase activity was normalized based on the total protein of each sample, measured using a commercial kit (Sigma-Aldrich, St. Louis, MO, USA; code TP0300), according to the manufacturer’s guidelines. Both rP and total protein were measured using an ELISA reader with a 630-nM filter (Polaris®, Celer Biotechnology, Belo Horizonte-MG, Brazil).

### Expression of Na/K-ATPase on the sperm surface

The method is a modification of that described by Oliveira et al. (2019). Two straws from each batch/bull were thawed, homogenized, and the total contents were split into two microtubes. The samples of the first microtubes were immediately processed, whereas the samples in the second microtube were maintained in a water-bath for 120 min (37°C) before evaluating the expression of Na/K-ATPase on the sperm surface.

To assess the expression of Na/K-ATPase on the sperm surface, the semen samples in each microtube were homogenized with 750 µL of modified TALP, followed by centrifugation (500 *g*, 5 min, room temperature) to remove the extender. After the supernatant was discarded, the sperm pellet was resuspended in 750 µL of modified TALP and homogenized. Subsequently, 2 µL of fluorochrome Bodipy^®^ FL Ouabain solution in PBS (FLOU; Sigma-Aldrich, St. Louis, MO, USA) was added to 198 µL of sperm samples and incubated (15 min, room temperature) to obtain 10^-7^ M of FLOU as final concentration.

After incubation with FLOU, the samples were analyzed by flow cytometry on a FACSCanto™ II device (BD Biosciences, San Jose, CA, USA) equipped with the FACS Diva software (Becton Dickinson). A gate excluding cell debris was determined using forward vs. side scatter parameters. Analyses were performed after recording 10,000 events for each sample, and fluorescence was determined in the FITC channel at 488 nm excitation and 525 nm emission. The flow cytometry results for the expression of Na/K-ATPase on the sperm surface of total cells were analyzed using the Flowing Software 2.5.1 and the results are expressed as the median of fluorescence intensity (Oliveira et al., 2019).

### Statistical analysis

Since the fertility classification of the bulls was determined by an institution specialized in bovine reproduction and based on an expressive number of reproductive data (at least 500 FTAI per bull, based on the Concept Plus program designed by Alta Genetics), eight animals and 24 seminal batches were considered satisfactory to perform the experiments. The normal distribution of the data was assessed by a Shapiro-Wilk test; each bull was considered an experimental unit (HF = 4 and LF = 4). For each analysis performed, three semen batches from each bull were used to assess the mean ± SE per bull, totalizing 24 ejaculates. The variables used for comparison were bull fertility and incubation time (0 and 120 min), when applicable. All analyses were carried out using the Past software (Version 4.01) and no data were transformed prior to the analyses. Differences between means in sperm kinematics, membrane integrity, and Na/K-ATPase expression on the sperm surface were assessed using two-way ANOVA, followed by a Tukey’s post-hoc test. The Na/K-ATPase enzymatic activities between HF and LF bulls were compared using the Mann-Whitney test. For all analyses, P < 0.05 was considered significant.

### Ethics 

Ethical approval is not required since this study was conducted with biological samples available commercially, according with Law 11794/2008 and Resolution 30/2016, from the National Council for the Control of Animal Experimentation (CONCEA), Brazil.

## Results

Within the same evaluation time (0 and 120 min post-thaw), there was no difference in sperm kinematics between HF and LF bulls (P > 0.05, Table 1), as well no difference between HF and LF bull samples for sperm membrane integrity.

**Table 1:** Kinematic of thawed sperm from Angus bulls scored as high (HF) and low fertility (LF), immediately after thawing (0 h) and after 2 h of incubation at 37°C.

**Parameter**	**Bull fertility and incubation time**			
	**HF**	**LF**	**HF**	**LF**
	**0 h**	**0 h**	**2 h**	**2 h**
TM (%)	77.9 ± 3.1^a^	78.8 ± 3.0^a^	48.1 ± 5.6^b^	57.2 ± 4.0^b^
PM (%)	46.6 ± 3.0^a^	44.7 ± 2.7^a^	29.0 ± 5.2^b^	31.1 ± 3.9^b^
VCL (µm/s)	67.9 ± 3.5^a^	59.3 ± 3.2^ab^	47.2 ± 3.2^c^	48.1 ± 2.6^bc^
VSL (µm/s)	36.0 ± 1.7^a^	32.6 ± 2.0^a^	22.1 ± 2.7^b^	21.6 ± 1.9^b^
VAP (µm/s)	46.4 ± 2.2^a^	41.8 ± 2.4^a^	29.2 ± 3.0^b^	29.0 ± 2.1^b^
LIN (%)	53.4 ± 1.5^ab^	55.0 ± 1.7^a^	45.3 ± 2.9^bc^	44.4 ± 2.4^c^
STR (%)	77.6 ± 1.0^a^	77.8 ± 1.1^a^	74.0 ± 1.9^a^	73.9 ± 2.0^a^
WOB (%)	68.6 ± 1.2^a^	70.6 ± 1.4^a^	60.5 ± 2.5^b^	59.7 ± 1.8^b^
ALH (µm)	2.6 ± 0.1^a^	2.3 ± 0.0^a^	2.3 ± 0.0^a^	2.5 ± 0.1^a^
BCF (Hz)	10.8 ± 0.3^a^	10.0 ± 0.2^a^	10.0 ± 0.6^a^	9.7 ± 0.6^a^

Within a row, means without a common superscript differed (P < 0.05).

The incubation time caused a significant reduction in most of the kinematic parameters, regardless of the bull fertility, except for STR, BCF, and ALH, which were not influenced by incubation time. It should be noted that in the samples from HF bulls, there was also no reduction in LIN after incubation time, and a similar pattern was observed for VCL in LF bulls (Table 1). Incubation also reduced (P < 0.05) sperm membrane integrity in samples from both HF and LF bulls (63.6 ± 2.2 and 69.3 ± 2.4 at 0 h; 54.2 ± 2.4 and 52.5 ± 1.8 at 2 h, respectively).

**Figure 1:**
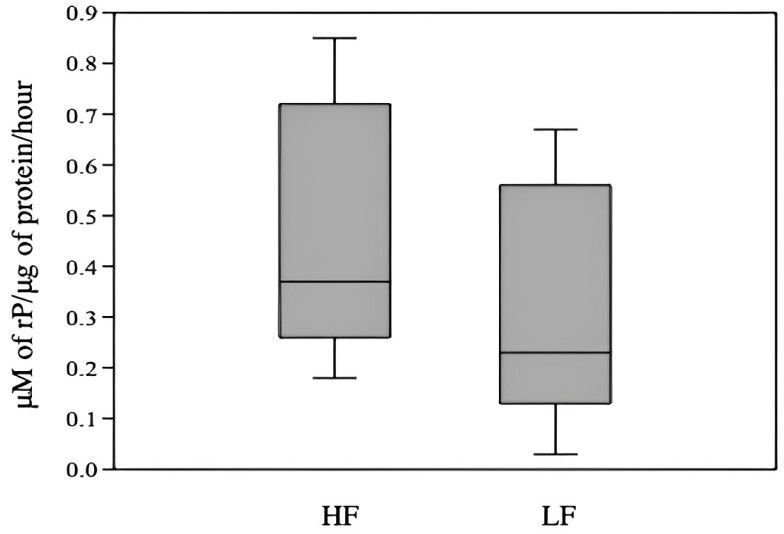
Box plot of Na/K-ATPase activity of thawed sperm from Angus bulls scored as high (HF) and low fertility (LF). P = 0.06.

**Figure 2:**
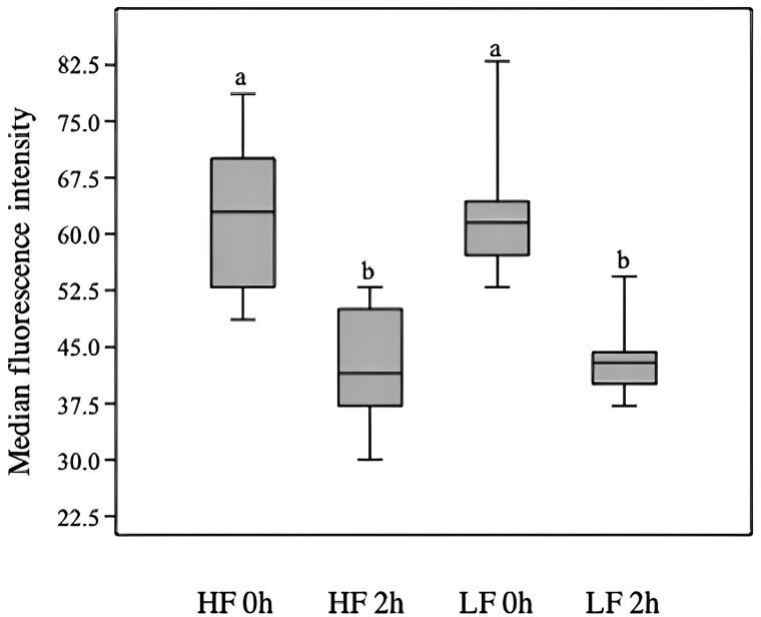
Box plot of Na^/^K-ATPase expression on surface of thawed sperm from Angus bulls scored as high (HF) and low fertility (LF), immediately after thawing (0 h) and after 2 h of incubation at 37°C. Means without a common superscript differed (P < 0.01).

The intra-assay coefficient of variation of Na/K-ATPase activity ranged from 7.4 to 8.6%, being observed a tendency (P = 0.06) of higher Na/K-ATPase activity for HF than LF bulls (0.49 ± 0.07 and 0.32 ± 0.06, respectively; Figure 1). Moreover, there was no influence of bull fertility on Na/K-ATPase expression on the sperm surface immediately after thawing nor after 120 min of incubation. However, the incubation time reduced (P < 0.001) the median fluorescence intensity in samples from both HF and LF bulls (Figure 2).

## Discussion

The similarity found between the samples of HF and LF bulls in terms of enzymatic activity and expression of Na/K-ATPase on the sperm surface differs from the results in a similar study performed with Holstein bulls (Rajamanickam et al., 2017b), which demonstrated that high-fertility bulls have higher activity and content of the α4 subunit of Na/K-ATPase than bulls with lower fertility.

It is noteworthy that Rajamanickam et al. (2017b) subjected the semen samples to a percoll wash, and detected the expression of Na/K-ATPase on the sperm surface by flow cytometry only in membrane-intact sperm, using an anti-α4 antibody, whereas we measured the Na/K-ATPase activity and expression on the sperm surface of total sperm population, using a fluorochrome conjugated to ouabain, a specific ligand and inhibitor of Na/K-ATPase (Schoner and Scheiner-Bobis, 2007). Percoll wash selects sperm subpopulations with superior motility and membrane integrity (Henkel and Schill, 2003) and excludes sperm subpopulations that had suffered more extensive cryoinjuries (Morrell, 2006; Maree and Van Der Horst, 2013).

Moreover, in the study of Rajamanickam et al. (2017b) the HF bulls had increased tyrosine phosphorylation than LF bulls. Since regulation of Na/K-ATPase activity is observed during capacitation (Jimenez et al., 2012), and it was observed that capacitation increased Na/K-ATPase content and activity in bull sperm, apparently through mitochondrial translation (Rajamanickam et al., 2017a); further than differences between measurement technique, species, microenvironment, and reproductive strategies adopted that influence bull fertility (Bollwein and Malama, 2023; Kathiravan et al., 2011; Harstine et al., 2018), it is plausible that sperm pre-selection using percoll and assessment of sperm traits in a specific subpopulation (membrane-intact sperm) explain the differences between the report of Rajamanickam et al. (2017b) and the present data.

The similar sperm kinematics between HF and LF bulls within the same incubation time is supported by a previous study with beef bulls, where no correlation between fertility and sperm kinematics was detected after more than 4,000 FTAI (Pereira et al., 2021). Similarly, no difference in any kinematic parameter was detected for bulls classified as below average, average, and above average fertility (Kumaresan et al., 2017); whereas in another study, a correlation was detected only between WOB and 56-day non-return rate (Morrell et al., 2018). Sperm membrane integrity did not differ between HF and LF bulls within the same time, which agrees with Al Naib et al. (2011), Gliozzi et al. (2017) e Rajamanickam et al. (2017b), who also reported no difference in sperm membrane integrity between bulls with different fertility levels. However, a relationship between membrane integrity and fertility is commonly reported (Christensen et al., 2011; Kumaresan et al., 2017). Those discrepancies highlight the importance of associating several sperm traits to better predict a sire's fertility (Kumaresan et al., 2017; Bollwein and Malama, 2023; Muhammad Aslam et al., 2014).

The incubation time (120 min) allowed identifying differences in the kinematic patterns of VCL and LIN for HF and LF bulls, respectively, which were not detected immediately post-thaw (0 min). It is well known that cryopreservation causes sublethal sperm freezing damage (Holt, 2000), inducing cryocapacitation/hyperactivation (Töpfer-Petersen et al., 2005), which can be characterized by high VCL and ALH values associated to low LIN and STR values (Muiño et al., 2008). Since the STR, ALH, and BCF values were similar throughout the incubation time, regardless of bull fertility (HF or LF), the kinematic pattern observed in LF bulls throughout the incubation time (maintaining VCL and reducing LIN values) might reflect the hyperactivation in sperm subpopulations. Conversely, the high LIN and reduction in VCL of the HF bull’s sperm after incubation indicates a lower percentage of hyperactivated sperm subpopulations, which in agreement with previous studies reporting that individual sperm velocities can be associated with the fertilizing potential of thawed sperm (Byrd et al., 1990; Krause, 1995).

The incubation time also reduced the surface expression of Na/K-ATPase in both HF and LF bull’s sperm. These findings may be related to the increasing of membrane-damage sperm during incubation, since the percentage of ram sperm expressing the Na/K-ATPAse reduced after cryopreservation, but no difference in median fluorescence intensity for Na/K-ATPAse in membrane-intact sperm was detected between fresh and thawed samples (Oliveira et al., 2019). Changes in the lipid configuration of the sperm membrane caused by cryopreservation (Giraud et al., 2000) can consequently affect the expression and activity of Na/K-ATPase by interfering with cholesterol stability (Chen et al., 2011) and the binding site of the Na/K-ATPase to the cell membrane (Shinoda et al., 2009). Thus, we infer that sublethal damage and sperm membrane modifications probably influenced the expression and/or integrity of Na/K-ATPase after the incubation period.

## Conclusion

In conclusion, we have provided evidence that Na/K-ATPase activity and expression in thawed sperm from Angus bulls is not related to fertility when total sperm population is evaluated. However, after sperm incubation, high-fertility bulls had a lower percentage of cells with hyperactivation-like kinematics, compared with low-fertility ones.
